# Heat Treatment of Corrosion Resistant Steel for Water Propellers Fabricated by Direct Laser Deposition

**DOI:** 10.3390/ma13122738

**Published:** 2020-06-17

**Authors:** Ruslan Mendagaliev, Olga Klimova-Korsmik, Vladimir Promakhov, Nikita Schulz, Alexander Zhukov, Viktor Klimenko, Andrey Olisov

**Affiliations:** 1 Institute of Laser and Welding Technologies, St. Petersburg State Marine Technical University, St. Petersburg 190121, Russia; ruslanm888@mail.ru (R.M.); o.klimova@ltc.ru (O.K.-K.); 2 Institute of Metallurgy, Mechanical Engineering and Transport, Peter the Great St. Petersburg Polytechnic University, St. Petersburg 195251, Russia; 3 SEC Siberian Center for Industrial Design and Prototyping, National Research Tomsk State University, Tomsk Oblast 634050, Russia; schulznikita97@gmail.com (N.S.); zhuk_77@mail.ru (A.Z.); klimenko@siberia.design (V.K.); science@klimenko.team (A.O.)

**Keywords:** direct laser deposition (DLD), direct metal deposition, additive manufacturing (AM), corrosion resistant steel, heat treatment (HT), maraging steel, microstructure, mechanical characteristics

## Abstract

The urgency of heat treatment of samples of maraging steel obtained by direct laser deposition from steel powder 06Cr15Ni4CuMo is considered. The structural features and properties of 06Cr15Ni4CuMo steel samples after direct laser deposition and heat treatment are studied. The work is devoted to research into the influence of thermal processing on the formation of structure and the mechanical properties of deposit samples. Features of formation of microstructural components by means of optical microscopy are investigated. Tests for tension and impact toughness are conducted. As a result, it was established that the material obtained by the direct laser deposition method in its initial state significantly exceeds the strength characteristics of heat treatment castings of similar chemical composition, but is inferior to it in terms of impact toughness and relative elongation. The increase in relative elongation and impact toughness up to the level of cast material in the deposit samples is achieved at the subsequent heat treatment, which leads to the formation of the structure of tempered martensite and reduction in its content at two-stage tempering in the structure of the metal. The strength of the material is also reduced to the level of cast metal.

## 1. Introduction

Currently, to increase the competitiveness of shipyards for the manufacture of parts of marine engineering, new high-tech technologies are used. Additive manufacturing methods are increasingly being used, including direct laser deposition technology (DLD). In the DLD process it is possible to obtain parts, including from shipbuilding steels used in the Arctic. Iron and its modified alloys are the most important class of metallic materials used in shipbuilding. Different grades of stainless steels can be treated as a part of traditional manufacturing techniques such as casting, machining, powder metallurgy and welding, including any combination of those. [1,2].

As a result of the research on estimation of material characteristics in 1980, this has been developed and mastered in the industry of martensitic–austenitic stainless steel (CA6NM—06Cr15Ni4CuMo) for manufacturing compressors [3], propeller blade [4,5,6,7], castings of blades, components of chemical and oil industry and other cast details of responsible purpose, and now for the manufacture of large castings for a propeller blade of blades and hub steel of 06Cr15Ni4CuMo. Currently, it is relevant to obtain parts and blanks for industrial production of this steel by the DLD method.

With the development of modern production technologies, it has become possible to manufacture parts from virtually any metal powder using additive technologies (AM). AM is characterized by quick fabrication and economical spending of expensive materials. Direct laser deposition (DLD) methods are of special interest when large workpieces need to be made using AM. The DLD technology makes it possible to fabricate large parts from stainless and cold-resistant steels [8,9,10,11,12]. The features of the DLD process include high temperature gradients, and repeated fast heating and fast cooling that cause residual strains and form heterogeneities in the microstructure. Microstructural features, such as grain size and morphology (as well as phase transitions), are very sensitive to the dynamic thermal history and they directly influence the microhardness, tear strength and modulus of resilience [13,14,15,16,17,18].

The mechanical properties of alloys obtained using DLD depend on their structure-phase states. For steels, an important role is played by the content of laminar low-carbon martensite (α′) microstructure and the presence of other phases, such as delta ferrite (δ), austenite (γ) and chromium carbides. It is known that the austenite phase is preserved in the tempering process and/or it is restored as a result of the treatment of quenched material. The secondary phase of ferrite in the alloy is δ-ferrite, which forms at very high temperatures during hardening in the course of casting or the DLD process [19].

To achieve high mechanical characteristics for martensite stainless steel, heat treatment (HT) is normally used. However, the microstructure of alloys obtained using DLD is close to a cast structure and is anisotropic. Such materials are characterized by low plasticity and modulus of resilience [20,21,22]. Here, the material’s inner structure is particularly influenced by cyclic heating during DLD due to layer-by-layer metal deposition [23,24,25]. During the fabrication process, the work piece is tempered. While classical casting and welding envisages a full HT cycle (quenching and tempering), parts fabricated by deposition require the development of ad-hoc HT that is different from the classical one [26,27]. The influence of these effects on the structural characteristics and mechanical properties needs research, specifically the presence of residual austenite γ, non-quenched martensite α′, and delta ferrite δ. These factors govern the final properties of the manufactured parts.

The goal of the research work was to grow a plate of 06Cr15Ni4CuMo steel and further reveal the patterns of microstructure formation and mechanical properties after high tempering and determine the maintenance regime providing the required level of ductility of steel not inferior to casting.

## 2. Materials and Methods 

### 2.1. Materials

**Figure 1:**
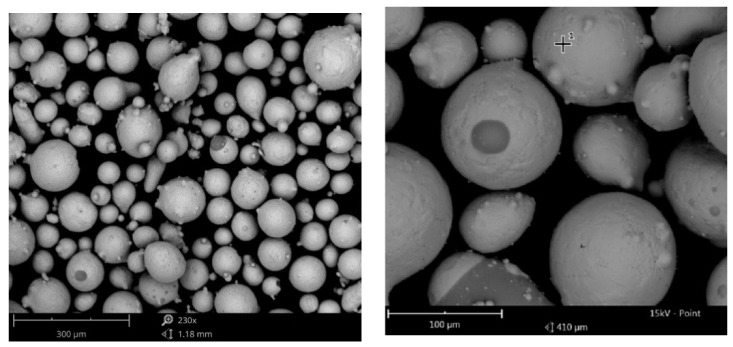
Surface of powder particles 06Cr15Ni4CuMo.

We have chosen 06Cr15Ni4CuMo (an analog of CA6NM) for the material. The starting material is 06Cr15Ni4CuMo fraction 45–160 µm Figure 1 is the producer of the “Polema” powder. The chemical composition of the steel is provided in Table 1.

The impact bending tests of the 06Cr15Ni4CuMo steel were conducted on an RKP 450 (Zwick/Roell, Ulm, Germany) unit at −10 °С, with impact energy 150 J and tensile tests were performed on a Z100 (Zwick/Roell, Ulm, Germany) unit at room temperature. Samples made under mechanical tensile testing GOST 1497-84 (RU) and impact testing GOST 6996-66 (RU).

To show the structure, we used chemical etching with Kalling’s reagent in a solution (33 mL HCl + 33 mL of ethanol + 33 mL of H_2_O + 1.5 g of CuCl_2_) over 30–60 s.

The deposited cladding layers were visually examined and instrumentally measured; then, they were investigated by optical microscopy on the DMI 500 Leica (Leica Microsystems, Wetzlar, Germany) microscopes using Thixomet (Thixomet, St.-Petersburg, Russia).

### 2.2. Fabrication of Samples Using DLD and Their Heat Treatment

**Figure 2:**
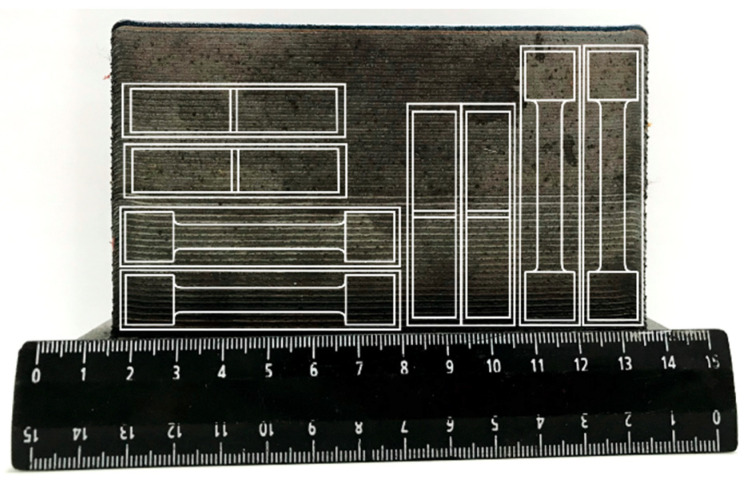
The scheme of cutting samples for mechanical testing.

We used the following equipment: a robotic complex based on an LRM-200iD_7L (Fanuc, Oshino, Japan) industrial robot; fiber laser based on a LS-3 Yb (IRE Polus Ltd., Fryazino, Moscow Region, Russia) unit; an FLW D30 (IPG Photonics, Oxford, UK) laser deposition head with a detachable SO12 (Fraunhofer IWS, Aachen, Germany) deposition nozzle and a Twin 10C (Sulzer Metco Inc., New York, NY, USA) powder feeder. A shielding gas atmosphere was used for deposition: an air-proof chamber filled with argon at an excess pressure of 2–3 MPa. In the argon-filled chamber the content of oxygen was not more than 300 ppm. Manufacturing of samples by DLD method was carried out at power P = 2300 W, speed V = 25 mm/s and cross section of depositing bead 2 × 0.8 mm^2^ powder flow rate G = 35 g/min, displacement along the Δx = 1.6 mm, and Δz = 0.7 mm. Five blocks (1 in the initial state and 4 per HT) with LxWxH dimensions 130 × 80 × 16 mm in Figure 2 were deposit simultaneously: a layer was applied alternately to each of the samples, after that the transition to the next layer took place.

DLD was carried out in a shielding chamber with controllable atmosphere. High purity argon was used as a transport and protective gas. Overpressure of 2–3 Mbar was maintained in the deposition chamber. The residual oxygen content in the working atmosphere did not exceed 300 ppm. The porosity in the grown plates did not exceed 2% of the total volume of the deposit sample.

**Figure 3:**
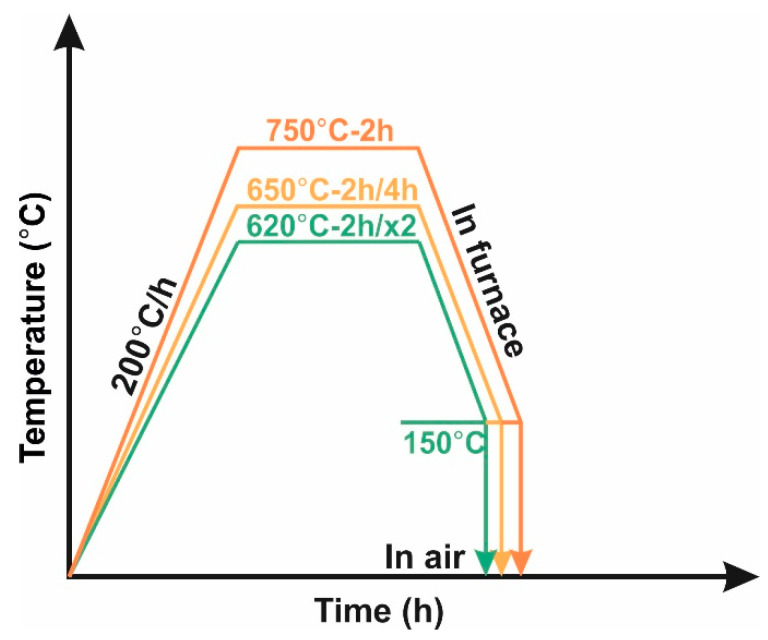
The heat treatment of the samples fabricated by deposition.

The heat treatment was performed in a SNOL 30/1300 muffle furnace without, shielding gas. The heating rate was 200 °С per hour with subsequent exposure and cooling as shown in Figure 3. Cooling rate of samples after high-temperature tempering was 50 °C per hour and then air cooling was 150 °C.

## 3. Results

In the course of parts fabrication from martensite grade steels using DLD, forced process thermal cycling is taking place due to extensive heat deposition. Heat deposition then impacts the presence of retained austenite and δ-ferrite [28] in the structure of the samples fabricated by DLD. Residual austenite can have a negative effect on hardness and toughness.

In the course of tempering during subsequent cooling, metal plasticity deteriorates, and this is due to the formation of secondary martensite as a result of conversion of residual austenite. That is why it makes sense to conduct a second tempering of secondary martensite. This promotes an increase in the metal’s relative elongation, creating a finer structure as a result of the decomposition of secondary martensite and the formation of quenched martensite.

Cast metal needs HT for quenching and dual subsequent tempering. During DLD, however, forced thermal cycling is taking place. This strengthens the samples, so we only need to conduct dual tempering to achieve the desired results. Based on the mentioned data from the literature and the results of experimental studies of the steel’s characteristics, we have found HT modes for the samples. These modes envisaged high-temperature tempering that would provide the best combination of mechanical properties: high strength, elongation, and impact strength [27].

After DLD the steel has high strength characteristics and low plasticity. To achieve the required mechanical properties for the steel, we have selected several HT modes, as shown in Table 2.

**Figure 4:**
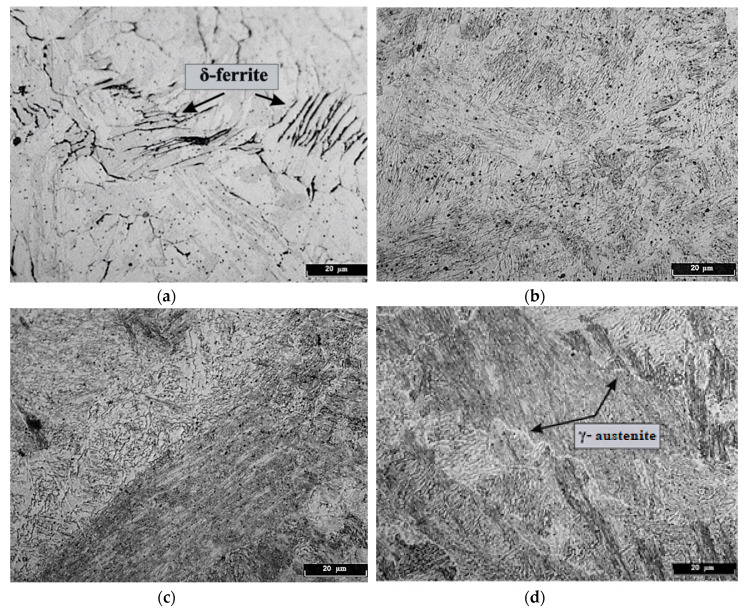

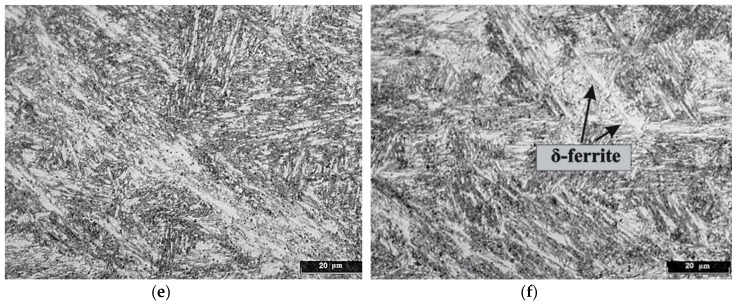
The microstructure of 06Cr15Ni4CuMo (**a**) DLD; (**b**) mode 1 (Т = 750 °С, t = 2 h); (**c**) mode 2 (Т = 650 °С, t = 2 h); (**d**) mode 3 (Т = 650 °С, t = 4 h); (**e**) mode 4 (Т = 620 °С, t = 2 h); (**f**) mode 5 (Т = 620 °С, t = 2 h/x2).

Different δ-morphologies were clearly revealed after etching the fabricated sample, Figure 4a. These morphologies are created by the incomplete growth of Widmanstetten γ-grains during the solid-state δ→γ phase transformation, Figure 4b. Here, incomplete growth results in residual δ stringer inclusions being left at the borders [4]. These inclusions outline the growth front that resembles the initial orientation of the ex- Widmanstett patterns in the final microstructure. During further cooling, γ-austenite converts into α′-martensite, but some amount of δ-delta-ferrite remains in the final microstructure, as shown in Figure 4a. Afterwards, the fabrication δ-delta-ferrite was discovered in the samples, and its content did not exceed 5%.

At such high temperatures, δ-grains are growing rapidly during heating. Then, during the cooling process, they convert into a γ-phase with subsequent transition into a α′ structure. In Figure 4b, after a single iteration of high-temperature tempering, some amount of γ-austenite (as well as M_7_C_3_ chromium carbides) is still observed.

Figure 4c,d shows some amount of non-converted α′-martensite that is an unstable microstructure in the α matrix. It promotes the formation of carbides, interlayer boundaries at large angles and a γ-phase.

Figure 4e,f includes a structure after high-temperature tempering that is represented by quenched lath α′-martensite with fine particles of residual austenite. The particles are located between martensite laths and at the boundaries of martensite batches with large inclusions of δ-ferrite in the matrix basis.

The best mechanical properties were achieved at dual tempering at Т = 620 °С, t = 2 h/x2 the cycle is repeated twice Figure 4. This mode complies with the technical specifications for this steel grade while having slightly lower plasticity characteristics.

**Figure 5:**
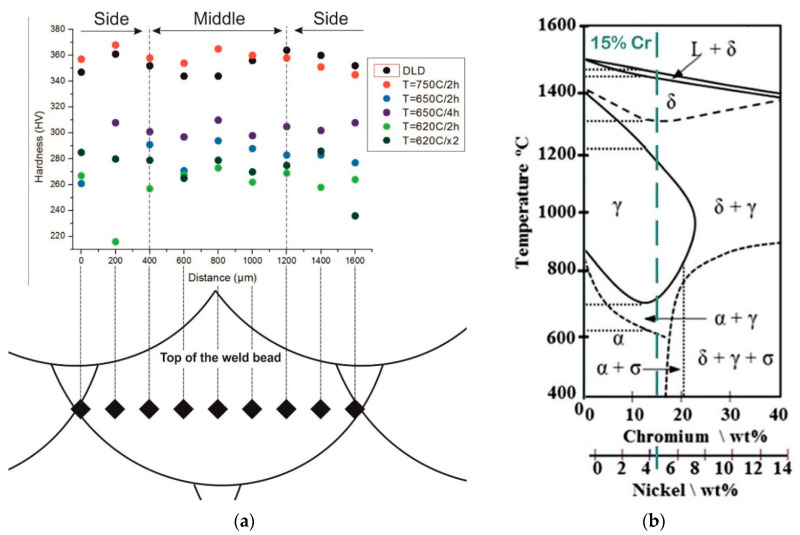
(**a**) microhardness; (**b**) the Fe–Cr–Ni equilibrium phase diagram [29].

Figure 5 shows the distribution of microhardness single weld bed and their arrangement for different HT modes and a thermokinetic diagram for Fe-Cr-Ni.

The average microhardness in the fabricated sample tempered at 750 °С, Figure 5b, was 355 HV. Tempering at 750 °С was chosen because of the phase transition into the γ-austenity area that preceded the dissolution of existing chromium carbides in the fabricated sample (i.e., M_23_C_6_/M_7_C_3_). Thus, it increased the concentration of carbon in the martensite matrix at room temperature.

With temperature reduction to 650 °С it was possible to decrease the hardness to 280 HV with an exposure time of 2 h, and an exposure time of 4 h was required to decrease it to 301 HV. At high-temperature tempering at Т = 620 °С with an exposure time of 2 h, the microhardness was 260 HV, and it was 273 HV after the second tempering.

## 4. Discussion

It is established that the DLD process is the fastest and most convenient for creating parts. After the process, it is necessary to produce high tempering to achieve all the necessary mechanical properties. Samples obtained by the DLD method are not inferior in characteristics to casting, and in some cases are most in demand.

Thus, we can conclude that the propeller with an optimized structure has reliability characteristics close to the original solid version. We showed the manufacturing of hub and blades via DLD and built-up propeller before and after CNC-machining and manual polishing. After all producing stages, the propeller was weighted. The weight test showed that the mass is finished product is 105 kg. This is 20% less than the original cast design. A more detailed description of design analysis, optimization procedure and production process is presented in [30].

## 5. Conclusions

The DLD process of 06Cr15Ni4CuMo steel achieves a high strength due to the forced thermal cycling process at low impact toughness and relative elongation. In order to eliminate the imbalance of the complex of mechanical properties, HT is proposed.

According to the results of a comparison of mechanical properties, it was established that the lowest structural matrices are tempered by fine martensite with a low level of residual austenite and δ-ferrite. Based on the analysis of the relationship between HT, mechanical properties and 06Cr15Ni4CuMo steel structure, the most suitable heat treatment mode for deposit samples was established, consisting of a double HT at Т = 620 °С, t = 2 h/x2; the cycle is repeated twice, in which a finely dispersed structure of tempered rack martensite is formed, providing a set of properties equal to the material obtained by casting.

## Figures and Tables

**Table 1:** The chemical composition of the steel.

Material Grade	Elements Mass Ratio, %									
	C	Si	Mn	Cr	Ni	Mo	S	P	Fe	Cu
06Cr15Ni4CuMo	≤0.06	0.40	0.60–0.90	14.0–15.5	4.0–4.4	0.11–0.28	0.015	0.015	Bal.	1.0–1.5

**Table 2:** Mechanical properties for heat treatment (HT) modes.

	P (W)	V (mm/s)	Yield Strength, σ_в_, (MPa)	Ultimate Strength, σ_0.2_, (MPa)	Relative Elongation, δ_s_, (%)	Impact Toughness, KV^−10^, (J)
Technical specifications	N/A	N/A	≥790	≥620	≥19	≥40
DLD	2300	25	1088	792	8	17
b/Т = 750 °С, t = 2 h						
Mode 1	1840	20	1114	798	7.5	16
c/Т = 650 °С, t = 2 h						
Mode 2	1840	20	863	530	15	39
d/Т = 650 °С, t = 4 h						
Mode 3	2300	25	891.7	587.2	12	29
e/Т = 620 °С, t = 2 h						
Mode 4	2300	20	816.4	698.3	16	42
f/Т = 620 °С, t = 2 h/x2 the cycle is repeated twice						
Mode 5	2300	20	804.4	666.8	19	42
